# Emerging Roles of ncRNAs in Type 2 Diabetes Mellitus: From Mechanisms to Drug Discovery

**DOI:** 10.3390/biom14111364

**Published:** 2024-10-27

**Authors:** Yue Yang, Hao Cheng

**Affiliations:** 1 State Key Laboratory of Natural Medicines, Jiangsu Key Laboratory of Druggability of Biopharmaceuticals, School of Life Science and Technology, China Pharmaceutical University, Nanjing 210009, China; 2 State Key Laboratory of Natural Medicines, Department of Pharmaceutics, China Pharmaceutical University, Nanjing 210009, China

**Keywords:** ncRNAs, T2DM, regulatory mechanism, drug discovery, ncRNA drug delivery

## Abstract

Type 2 diabetes mellitus (T2DM), a high-incidence chronic metabolic disorder, has emerged as a global health issue, where most patients need lifelong medication. Gaining insights into molecular mechanisms involved in T2DM development is expected to provide novel strategies for clinical prevention and treatment. Growing evidence validates that non-coding RNAs (ncRNAs) including microRNAs (miRNAs), long non-coding RNAs (lncRNAs), and circular RNAs (circRNAs) function as crucial regulators in multiple biological processes of T2DM, inspiring various potential targets and drug candidates. In this review, we summarize the current understanding of ncRNA roles in T2DM and discuss the potential use of ncRNAs as targets and active molecules for drug discovery.

## 1. Introduction

According to the latest data issued by the International Diabetes Federation (IDF), the global prevalence of diabetes reached 10.5% in 2021. A total of 537 million adults were living with diabetes, representing a 16% increase (74 million cases) compared to 2019. Nevertheless, nearly half (44.7%) of adults remained undiagnosed. The IDF predicts that by 2045, the number of adult cases of diabetes will reach 784 million, exceeding 20% of the current estimated total adult population [1,2]. Diabetes has emerged as another significant disease that severely impacts people’s health following cardiovascular and cerebrovascular diseases [3,4]. Diabetes is a chronic disorder characterized by hyperglycemia, which results from relative or absolute insulin deficiency, reduced sensitivity of target cells to insulin, and disturbances in glucose, lipid, and protein metabolism [5]. Diabetic patients typically present four metabolic abnormalities: abnormal insulin action, dysfunctional insulin secretion, increased endogenous glucose output, and obesity [6,7,8]. Currently, the high mortality rate of diabetes poses a severe threat to both individuals and society. This disease renders a large number of people susceptible to fatal complications, such as heart disease and other diabetes-related disorders [9]. Over 90% of diabetes patients have type 2 diabetes (T2DM) [10]. Sedentary lifestyles, obesity, aging, and urbanization are contributing factors for T2DM. Additionally, genetic background is also implicated in the etiology of this disease. The hallmark of T2DM is chronic hyperglycemia and abnormal metabolism of carbohydrates, lipids, and proteins, mainly attributed to insulin resistance and insufficient insulin secretion. The interaction between genes and lifestyles further complicates the etiology of diabetes [11,12].

The regulation of type 2 diabetes is a complex network, involving multi-level and multifactorial interactions, including insulin receptors and proteins in insulin signaling pathways. For instance, the PI3K/AKT signaling pathway is required for normal metabolism because the pathway plays a key role in regulating glucose metabolism, and its imbalance leads to the development of obesity and T2DM [13,14,15]. At the same time, some small molecules affect insulin sensitivity and secretion through metabolic pathways, such as fatty acids, glucose, and insulin [16,17,18,19]. The epigenome includes DNA methylation, histone modifications, and RNA-mediated processes, and disruption of this balance may cause several pathologies and contribute to obesity and T2DM [20,21]. Furthermore, the regulation of functional non-coding RNAs (ncRNAs) can alter gene activity, which modulates gene expression and transcription, chromatin structure, epigenetic memory, selective RNA splicing, and protein translation [22,23,24].

NcRNA is a type of functional RNA molecule that is typically not translated into proteins and constitutes 98% of the human genome. In the early days, ncRNA was regarded as the “dark matter” in the process of gene transcription and was considered non-functional. However, with the advancement of technology in recent years, it has been discovered that ncRNA plays an extremely significant role in the occurrence and development of diseases. ncRNA has numerous classifications, including transfer RNA (tRNA), ribosomal RNA (rRNA), small nuclear RNA (snRNA), small interfering RNA (siRNA), microRNA (miRNA), piwi-interacting RNA (piRNA), long non-coding RNA (lncRNA), and circular RNA (circRNA) [25]. Among them, functional ncRNAs can be further categorized into three types: long non-coding RNA (>200 nucleotides (nt)), microRNA (<200 nt), and circular RNA (with a circular structure), which regulate cellular processes and exert biological functions through direct interactions with each other [26]. For instance, miRNA can inhibit gene expression by targeting mRNA or regulate the stability of circRNA and lncRNA through targeted binding. On the other hand, circRNA and lncRNA can serve as “sponges” to modulate the expression level or functional domains of miRNA. Simultaneously, long ncRNA can regulate the physiological functions of cells or the occurrence and development of diseases by interacting with DNA or proteins [27,28,29,30]. Abnormal expression or interaction of ncRNA may result in the development of various diseases, including metabolic and cardiovascular disorders. A growing body of evidence indicates that miRNA, lncRNA, and circRNA are extensively involved in the pathogenesis of type 2 diabetes and act as key nodes in the disease regulatory network, providing novel targets for disease treatment and new drug development [26]. This review summarizes recent research on the regulatory mechanisms of ncRNAs in T2DM and discusses the significance of ncRNA as diagnostic and therapeutic targets for type 2 diabetes, aiming to offer references for the study of the pathogenesis and regulatory mechanisms of type 2 diabetes as well as the development of new clinical drugs and diagnostic strategies.

## 2. miRNA in T2DM

MicroRNAs (miRNAs) are a category of non-coding RNAs averaging 22 nucleotides, and their discovery has profoundly influenced the advancement of modern disease-related molecular biology [31]. The high conservation of miRNAs among different species and that of their targets indicate their critical role in the fundamental aspects of gene regulation. The dysregulated expression of miRNAs is associated with multiple diseases, among which T2DM is included. miRNAs play a significant role in the pathophysiology of T2DM, both intracellularly and extracellularly [22,23,32,33,34]. Notably, miRNAs can be secreted from specific cells or tissues into extracellular body fluids such as serum, plasma, saliva, or milk [35]. These miRNAs are typically present in exosomes, exosome-like vesicles, or microvesicles. Over the past few years, significant attention has been given to the characteristics of miRNAs in the pathophysiology of diabetes, particularly in T2DM, and numerous studies have been conducted [36]. Recent research indicates that miRNAs play a crucial role in modifying cellular pathways, namely, β cell survival, function, and peripheral insulin signaling, which are related to the onset and development of diabetes (primarily T2DM) [37,38,39]. This underlines the potential of miRNAs as diagnostic biomarkers and therapeutic targets for a variety of diseases.

### 2.1. Biosynthesis and Biofunctions of miRNAs

The biogenesis of miRNA encompasses several crucial steps. (1) Transcription by RNA polymerase II: miRNA genes are initially transcribed by RNA polymerase II into primary miRNA (pri-miRNA) transcripts, which typically range from approximately 500 to 3000 nucleotides in length. These pri-miRNAs commonly possess characteristic miRNA hairpin structures. (2) Intranuclear processing: pri-miRNA undergoes processing within the nucleus. This processing is executed by the microprocessor complex, which comprises the endonuclease Drosha and its cofactor DGCR8 (DiGeorge syndrome critical region protein). The microprocessor complex cleaves pri-miRNA into pre-miRNA of approximately 70 nucleotides and subsequently exports it from the nucleus to the cytoplasm. (3) Cytoplasmic trafficking: Exportin-5, a transport protein, in conjunction with the Ran GTP-binding protein, mediates the translocation of pre-miRNA across the nuclear membrane into the cytoplasm. (4) Dicer-mediated cytoplasmic processing: Within the cytoplasm, pre-miRNA is further processed into mature miRNA. This step involves the endonuclease Dicer, which truncates pre-miRNA into a double-stranded RNA duplex, with each chain approximately 22 nucleotides in length. One of the chains within this duplex is typically unstable and undergoes degradation, while the other chain functions as the mature miRNA and is incorporated into the RNA-induced silencing complex (RISC). (5) Enrichment in RISC and gene regulation: Mature miRNA binds to RISC, thereby mediating post-transcriptional regulation of gene expression. This regulation can be accomplished by inhibiting translation or promoting mRNA degradation and is contingent upon the degree of complementarity between miRNA and its target mRNA [40,41,42,43,44].

**Figure 1:**
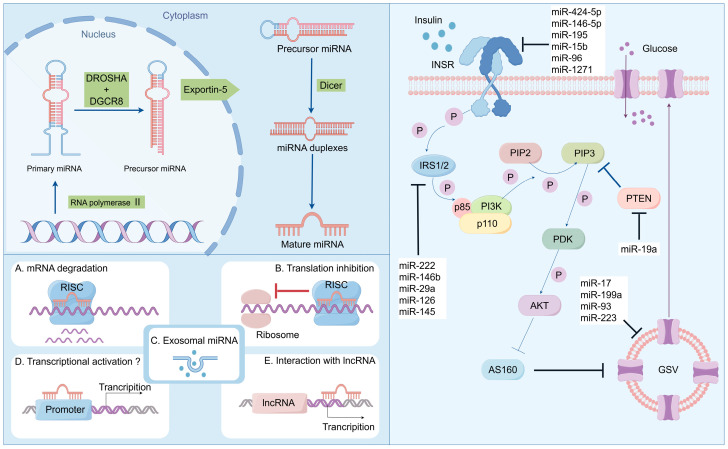
Biosynthesis and function of miRNA: regulating the disease progression of insulin resistance and T2DM.

miRNAs participate in numerous biological processes, encompassing development, differentiation, and environmental responses. miRNAs can modulate cellular pathways by either activating or inhibiting genes related to the pathway or by targeting the promoter region of the gene for activation. miRNAs can also alleviate gene expression by controlling translational expression and degrading the corresponding messenger RNA [45]. According to Bartell, miRNAs act as a guide for RISCs, which partially or completely splices the mRNA. On numerous occasions, in animals, the 7-nucleotide sequence from positions 2 to 7 in miRNAs starting from the 5′ end, which is designated as the seed sequence, binds to the 3′ end UTR of the target mRNA to guide the RISCs [43,46]. This process of gene suppression or activation induced by various miRNAs is associated with many diseases and is a factor contributing to the etiology and pathogenesis of these diseases (Figure 1).

### 2.2. miRNAs Modulate Pancreatic β Cell Functions

The function of pancreatic β cells and the insulin signaling (IS) pathway are intracellular metabolic signaling pathways related to maintaining glucose homeostasis in the body. Hence, alterations in IS and the survival and function of β cells play a significant role in impaired glucose homeostasis [47,48]. The process of insulin resistance mainly occurs in insulin-sensitive tissues, namely, the liver, muscle, and adipose tissue, mainly due to the dysregulation of the IS [49,50]. Nevertheless, there is evidence suggesting that insulin resistance in peripheral tissues increases the demand for insulin, ultimately augmenting the function of β cells [51]. Theoretically, the accumulation of insulin secretion by β cells alters glucose sensing, insulin synthesis, processing and secretion, proliferation, and survival of β cells, namely, β cell dysfunction. It is known that the dysfunction of these cells predominates in T2DM [37,52,53]. The decrease in β cell mass and the loss of normal function are potential pathogenic factors associated with pancreatic β cell dysfunction.

Recent studies have indicated that miRNAs play a vital role in the pathogenesis of DM (primarily T2DM) by dysregulating cellular signaling pathways. Some miRNAs that are highly expressed in pancreatic β cells and peripheral insulin-sensitive cells can suppress the translation of proteins in related pathways that are crucial for β cell survival, β cell function, and peripheral insulin sensitivity. Additionally, the specific miRNA profiles observed in the extracellular fluid of T2DM patients enable the investigation of potential miRNAs as biomarkers [23,33,34]. Poy and colleagues were the first to demonstrate the control of β cell activity by miRNAs. In this study, miR-375, a miRNA highly abundant in human pancreatic islets and mouse pancreatic islets, was reported to regulate the secretory activity of β cells [54,55]. This research indicated that silencing miR-375 would increase glucose-stimulated insulin secretion (GSIS) in the MIN6 mouse pancreatic β cell line and isolated primary β cells. The molecular mechanism is that miR-375 directly acts on myotrophic factor (Mtpn)/V-13’UTR to inhibit glucose-mediated insulin secretion in pancreatic beta cell line Min6 [56]. However, the role of miR-375 in glucose-regulated insulin secretion is not limited to the effects of muscle nutrient factor interactions. MiR-375 directly affects PDK1 mRNA and reduces its protein levels, leading to lower glucose stimulation and triggering insulin gene expression and DNA synthesis in beta cells [57]. Further research found that the level of circulating miR-375 significantly increased two weeks before the onset of diabetes, indicating that circulating miR-375 can be used as a marker of β cell death and a potential predictor of diabetes [58]. Another pancreatic β-cell-specific miRNA, miR-7a, belonging to the miR-7/7ab family, has been reported as a negative regulator of adult β cell proliferation by targeting multiple components of the mammalian target of the rapamycin (mTOR) signaling pathway. Wang and colleagues demonstrated in this study that the inhibition of miR-7a could activate the mTOR signaling pathway and promote β cell replication in mouse pancreatic islets, suggesting that miR-7a might be a potential therapeutic target for diabetes. Meanwhile, many miRNAs can regulate islet β-cell-related functions [59,60]. For instance, miR-486-5p and the miR-17-92 cluster regulate β cell proliferation by targeting phosphate and tensin homolog (PTEN) [61,62]; miR-375, miR-30a, and miR-34a exacerbate β cell damage [63,64]; and miR-130a-3p, miR-130b-3p, and miR-152-3p reduce the cytoplasmic adenosine triphosphate (ATP) level in the INS-1 cell line and inhibit insulin secretion by partially targeting pyruvate dehydrogenase alpha 1 (PDHA1) and Glucokinase (GCK) [65,66].

### 2.3. miRNAs Modulate Insulin Resistance

Insulin resistance (IR), also referred to as impaired insulin sensitivity, arises when cells in the muscles, fat deposits, and liver exhibit a lack of insulin response. Insulin is a hormone released by the pancreas that plays a vital role in maintaining blood glucose levels and ensuring survival. This condition can be temporary or chronic, with the potential for curability. Additionally, insulin resistance is characterized by diminished biological reactions to insulin stimuli within target tissues such as the liver, muscles, and adipose tissue. It obstructs proper glucose disposal and triggers an increase in beta-cell insulin production along with compensatory hyperinsulinemia. Numerous miRNAs have been implicated in diabetes development based on available evidence. Moreover, the dysregulation of miRNAs has been observed to induce insulin resistance across various target tissues, including the liver, skeletal muscle, and adipose tissue.

The role of miR-592 in the development of hyperglycemia and IR caused by obesity is significant. The target gene of this miRNA has been identified as FOXO1. It has been suggested that a reduced expression of miR-592 in the livers of obese humans and mice contributes to hyperglycemia and decreased sensitivity to insulin. Additionally, experiments using adenovirus and LNA in both obese and lean mice have demonstrated that increasing levels of hepatic miR-592 can effectively address various metabolic disorders associated with these conditions [67]. In HepG2 cell lines, the overexpression of miR-543 was found to decrease sirtuin 1 (SIRT1) levels under high glucose stress, leading to insulin resistance. Consequently, SIRT1 overexpression may be utilized as a countermeasure against the effects induced by miR-543 [68]. Furthermore, it was observed that diet-induced obesity led to an increased expression of miR-15b in HepG2 cell lines, resulting in the inhibition of insulin receptor (INSR) expression, a direct target gene regulated by miR-15b, ultimately leading to impaired insulin signaling and hepatocyte insulin resistance [66].

Adipose tissue, a significant endocrine organ involved in energy metabolism, releases various factors. miRNAs play a crucial role in regulating the differentiation and function of adipocytes. The harmful impact of elevated levels of miR-29 family (a, b, and c) on glucose absorption was observed due to the decreased expression of glucose transporter 4 (GLUT4). Direct targeting of secreted protein acid rich in cysteine (SPARC) by the miR-29 family was discovered [69]. Moreover, transfection with the miR-29 family reduced SPARC expression in 3T3-L1 cells. Consequently, the upregulation of the miR-29 family leads to reduced levels of SPARC protein and GLUT4, impairing glucose uptake by 3T3-L1 adipocytes [70]. The regulation of insulin sensitivity is controlled by two important miRNAs: miR-103 and miR-107 target caveolin-1. These two miRNAs were found to be upregulated in the liver of diet-induced obese mice, resulting in decreased glucose uptake. Conversely, silencing these two miRNAs increased caveolin-1 expression, improving insulin sensitivity and signaling while increasing blood glucose levels and reducing adipocyte size. In mouse models with obesity and individuals with fatty liver disease, it has been demonstrated that miR-107 suppresses fatty acid oxidation, establishing a connection between this specific microRNA and diabetes (Figure 1) [71,72,73].

Interestingly, miRNAs possess the potential to serve as highly efficacious therapeutic targets owing to their capacity to exert influence on the expression of a broad spectrum of target genes. However, further investigation into metabolic disorders is imperative in order to fully exploit the therapeutic advantages offered by miRNAs. It is anticipated that through additional exploration and functional characterization, the target genes associated with numerous miRNAs will be comprehensively elucidated. Moreover, by employing these findings in computational and experimental studies, it may be feasible to identify specific clinical biomarkers and develop effective interventions for managing insulin resistance and T2DM.

## 3. LncRNA in T2DM

**Figure 2:**
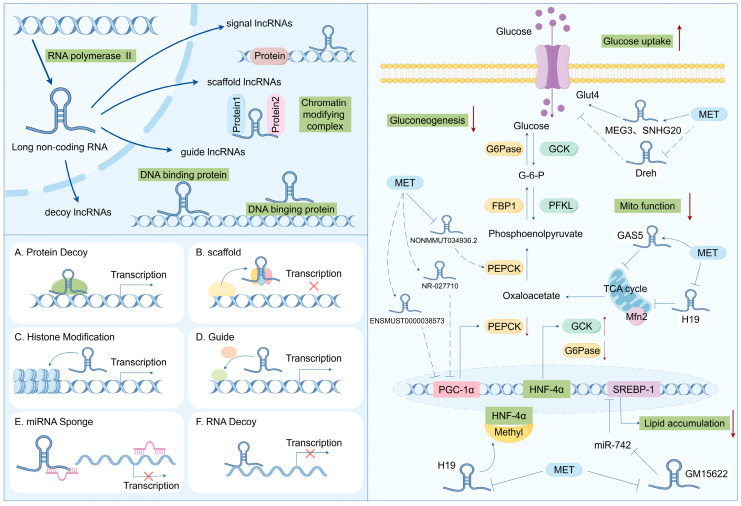
Biosynthesis and function of lncRNA: regulating the disease progression of insulin resistance and T2DM.

Long non-coding RNAs (lncRNAs) refer to transcripts longer than 200 nucleotides (nt), which are mainly located in regions of the genome with relatively low conservation and do not encode proteins [74]. Based on their genomic transcriptional locations, lncRNAs can be classified into seven types (Figure 2). Intergenic lncRNAs (intergenic lncRNAs), also known as lincRNAs, are lncRNAs transcribed from the intergenic regions between two coding genes and are at least 1 kb away from the coding genes. They primarily regulate cellular activities. Intronic lncRNAs (intronic lncRNAs) are mainly generated in the introns of coding genes, possess corresponding coding genes, and share the same expression patterns, and they mainly modulate gene expression. Antisense lncRNAs (antisense lncRNAs) are mainly produced in the antisense strand of the coding strand and bind to and regulate the expression of mRNAs. Sense lncRNAs (sense overlapping lncRNAs) have a transcriptional direction similar to that of adjacent mRNAs and partially or completely overlapping exons. They contain an open reading frame (ORF) for protein translation but do not encode proteins and limit the mRNA translation process by mediating the termination codon; promoter-associated lncRNAs and untranslated region-overlapping lncRNAs (untranslated region-overlapping lncRNAs) bind to the promoters and untranslated regions of regulated mRNAs. Enhancer subtype lncRNAs (enhancer lncRNAs) mainly regulate the expression of adjacent genes by binding to cis-regulatory sites or enhancers [75,76,77].

### 3.1. Biosynthesis and Biofunctions of lncRNAs

The biogenesis of lncRNAs is similar to that of mRNAs, and the majority of lncRNAs are transcribed by RNA polymerase II (RNAP2) in the nucleus from intergenic regions, introns of protein-coding genes, or the antisense strand of genes, but lncRNAs have some unique features that can distinguish them from mRNAs. For example, lncRNAs have relatively low expressions and the presence of fewer and longer exons than mRNAs [78,79]. Although they cannot produce proteins, they can act as pathway switches to participate in the regulation of signaling pathways. LncRNAs can function as scaffolds (bringing two distant genes closer), decoys (binding to transcription factors to inhibit gene transcription), guiders (guiding regulatory proteins to gene sequences), and genomic targeting (directly binding to gene sequences to affect gene expression), thus participating in molecular and genomic modulation. Meanwhile, a single lncRNA may have multiple targets, so its function may be diverse; additionally, lncRNAs do not conserve as many species compared to miRNAs, which makes the investigation of lncRNAs difficult [80].

Similar to the mRNA of protein-coding genes, lncRNA possesses a 5′cap and a 3′poly A tail. lncRNAs utilize the same genes as transcription templates and form distinct lncRNA transcripts through alternative splicing. In contrast to mRNA, lncRNA exhibits strong tissue specificity and has a lower abundance than mRNA [81]. It can mediate diverse biological processes through multiple mechanisms, namely, transcriptional interference, inducing chromatin remodeling, regulating alternative splicing patterns, modulating protein activity, and altering the intracellular localization of proteins. For instance, lncRNA can serve as a cis-acting element to regulate the expression of protein-coding genes in the vicinity of its own expression site. Some lncRNAs can also function as sponges for miRNAs, namely, competitive endogenous RNAs (ceRNAs). By binding to one or more miRNAs, they thereby regulate miRNA-mediated post-transcriptional silencing (Figure 2) [29,82,83]. These mechanisms accentuate the significant role of lncRNAs in various biological processes, and the dysregulation of lncRNA expression may lead to the advancement of various diseases, including T2DM.

### 3.2. LncRNAs Modulate Insulin Synthesis and Secretion in Pancreatic β Cells

Recently, researchers have uncovered that lncRNAs mediate biological processes in islet cells, such as β cell differentiation and proliferation, as well as insulin biosynthesis and secretion. Moran et al. identified 1128 high-confidence human islet cell genes through transcriptome sequencing, among which 55% were intergenic lncRNAs and 40% were antisense lncRNAs, and these genes were specifically expressed in the islets [84]. Ku et al. identified over 1000 long intergenic non-coding RNAs (lincRNAs) in mouse islets via RNA-seq, with the majority being β cell specific [85]. Recent studies have revealed that several long non-coding RNAs (lncRNAs) are capable of regulating insulin secretion and sensitivity. For instance, lncRNA metastasis-associated lung adenocarcinoma transcript 1 (lncMALAT1), acting as a negative regulator, interacts with nuclear factor erythroid 2-related factor 2 (Nrf2). The ablation of lncMALAT1 can activate the expression of Nrf2-regulated antioxidant genes and reduce ROS accumulation and oxidative stress, thereby diminishing inflammation, sensitivity to insulin signal transduction, and enhancing β cell function [86]. Xu et al. reported that SRA plays a positive role in promoting insulin sensitivity and insulin-stimulated glucose uptake. The mechanism is partially attributed to SRA’s ability to regulate the expression of multiple factors influencing insulin sensitivity. This encompasses, in ST2 adipocytes, SRA overexpression, which inhibits the expression of negative regulators of insulin sensitivity, such as cytokine signaling suppressor (SOCS)-1 and -3, while promoting the expression of positive regulators, such as SH3 domain-containing 1 (Sorbs1). Moreover, SRA also increases insulin-stimulated glucose uptake and potentiates the functional effects of insulin through the Akt and FOXO1 signaling pathways [87]. Another lncRNA abundantly present in the islets, lncRNA βFaar, has been reported to be involved in regulating insulin secretion and inhibiting apoptosis. Zhang et al. discovered that in both in vitro and in vivo conditions, the downregulation of βFaar increases apoptosis of islet β cells and reduces insulin secretion. The knockdown of lncRNA βFaar in Min6 cells leads to decreased mRNA levels of transcription factors related to insulin synthesis/secretion, such as neurogenic differentiation 1 (NeuroD1), cAMP response element-binding protein 1 (Creb 1), and insulin 1 [88]. To sum up, these studies imply the potential therapeutic roles of lncRNAs in enhancing insulin secretion and sensitivity (Figure 2).

### 3.3. LncRNAs Modulate Insulin Resistance

Insulin resistance is a crucial factor in the development of type 2 diabetes mellitus (T2DM), and long non-coding RNAs (lncRNAs) play significant roles in regulating insulin signaling and lipid metabolism. For instance, lncRNA MEG3 regulates insulin sensitivity by interacting with key proteins in the insulin signaling pathway. Furthermore, lncRNA HOTAIR influences insulin signaling by modulating the expression of insulin receptor substrate 1 (IRS1), thereby affecting insulin sensitivity in vivo [89].

Meanwhile, lncRNA has been recently discovered to act as a significant regulator in multiple biological processes, including adipocyte differentiation. There exists a strong correlation between adipocyte differentiation and insulin resistance. Throughout the process of adipocyte differentiation, precursor cells undergo transformation into mature adipocytes, facilitating fat storage. Excessive proliferation or enlargement of adipocytes can result in chronic inflammation and disruptions in cellular signaling, thereby instigating insulin resistance. Modulating the differentiation and functionality of adipocytes represents a crucial avenue for enhancing insulin sensitivity [90]. SRA (steroid receptor RNA activator) was initially identified as an lncRNA with regulatory functions in adipogenesis. Research indicates that SRA promotes 3T3-L1 adipocyte differentiation by binding to PPARγ and increasing its expression. It has also been found that other lncRNAs regulate the transcriptional activity of PPARγ, such as IMFNCR, ADNCR, and Plnc2. Similarly, several lncRNAs specifically modulate C/EBPα [91,92,93]. For instance, C/EBPα activates the expression of lncRNA TINCR by binding to its promoter, and TINCR adsorbs miR-31 to enhance the transcriptional activity of C/EBPα, forming a TINCR-miR-31-C/EBPα feedback loop and strengthening adipogenic differentiation in adipose tissue-derived mesenchymal stem cells (ADSCs) [94]. Additionally, Zhao et al. discovered a novel lncRNA, brown adipose lncRNA 1 (Blnc1), which forms a positive feedback ribonucleoprotein transcription complex with the transcription factor early B cell factor 2 (EBF2), guiding adipogenesis towards a thermogenic phenotype [95]. In conclusion, these studies suggest that lncRNAs play essential roles in regulating adipocyte differentiation and might offer novel insights for diabetes treatment (Figure 2).

## 4. CircRNA in T2DM

Over the past decade, circular RNAs (circRNAs) have emerged as a large class of primarily non-coding RNA molecules, many of which have key roles in diabetes development and progression through diverse mechanisms of action. CircRNAs often have tissue-restricted and specific expression patterns, and accumulating data suggest that these molecules are of potential clinical relevance and utility [96]. In particular, circRNAs have strong potential as diagnostic, prognostic, and predictive biomarkers, which is underscored by their detectability in liquid biopsy samples, such as in plasma, saliva, and urine [97]. CircRNA has great potential as a novel target or drug molecule for the intervention and treatment of T2DM.

### 4.1. Biosynthesis and Biofunctions of circRNAs

**Figure 3:**
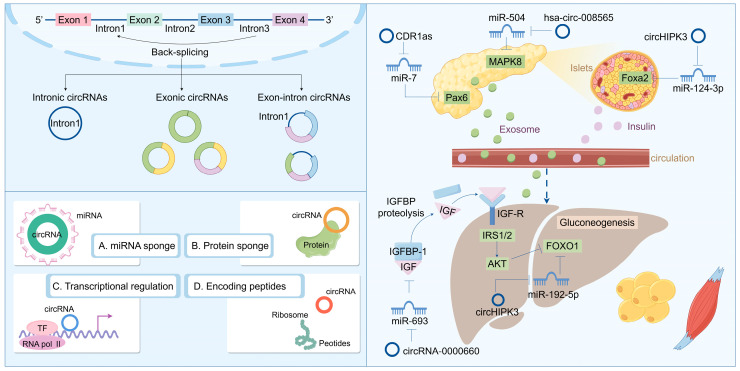
Biosynthesis and function of circRNA: regulating the disease progression of insulin resistance and T2DM.

Recent research into circRNA biogenesis has shown that back-splicing is catalyzed by the canonical spliceosomal machinery and modulated by both intronic complementary sequences (ICSs) and RNA-binding proteins (RBPs). Unlike linear RNAs, such as lncRNA and miRNA, circRNA is a covalently closed circular single-stranded RNA that typically lacks the capacity to encode proteins. The majority of downstream splicing sites are linked upstream of the 3′ splicing site. This procedure results in the formation of a circular RNA molecule with a 3′-5′ phosphodiester bond at the reverse splicing junction. Owing to the absence of a 5′ cap and a 3′ poly(A) tail, circRNA is immune to RNA exonucleases, thereby rendering its expression more stable and less susceptible to degradation. Nevertheless, as the efficiency of reverse splicing is significantly lower than that of conventional splicing, the abundance of circRNA in cells and tissues is typically low [98,99,100]. Based on its composition, circRNA can be categorized into several types: exonic circular RNA (ecircRNA) formed by exons; intronic circular RNA (ciRNA) formed by introns; and exonic-intronic circular RNA (EIciRNA) formed by both exons and introns. They typically exert functions at the transcriptional or post-transcriptional level. For instance, circRNA can serve as a sponge for miRNA, inhibiting the binding of miRNA to the non-coding regions of target genes, thereby regulating gene expression. They also interact with transcription factors to impact gene transcription. Additionally, circRNA interferes with the normal splicing of mRNA precursors, regulates mRNA translation, forms circular RNA–protein complexes, and competes with mRNA-binding proteins to participate in biological processes such as immunity, metabolism, and neural system development [101,102,103]. Some research indicates that circRNA plays a crucial role in the pathogenesis and development of diseases, particularly in T2DM, where it may influence blood glucose levels by regulating β cell proliferation and glucose metabolism (Figure 3). Regulating the expression levels of circRNA to maintain normal cellular functions might offer a novel approach to the treatment of T2DM.

### 4.2. CircRNAs Modulate Pancreatic β Cell Functions

Although current research on the regulatory mechanism of circRNAs is relatively limited, existing studies have already demonstrated that they play a significant role in the pathogenesis of T2DM. Numerous studies have indicated that circRNA plays a crucial role in the development, proliferation, and insulin secretion of pancreatic beta cells. For instance, circRNA_008565 is downregulated in T2DM rat models and binds to miR-504 to influence autophagy in rat pancreatic beta cells [104]. Moreover, the level of circ-Tulp4 in the islets of T2DM mice is decreased. Further studies have revealed that circ-Tulp4 regulates beta cell proliferation by targeting the miR-7222-3p/soat1/cyclin D1 signaling pathway. Another study using streptozotocin (STZ)-induced T2DM rat models has shown that circANKRD36 is highly expressed in T2DM rats, and silencing circANKRD36 can reduce blood glucose and inhibit insulin resistance and inflammation by targeting the miR-145/XBP1 axis [105]. In addition to the research findings in rats, some circRNAs have also been discovered to be related to T2DM in patients. A recent study identified twenty-one differentially expressed circRNAs in the venous blood of fasting T2DM patients, including five upregulated and sixteen downregulated circRNAs. Further analysis indicated that the downregulated circRNAs (circ_0016196, circ_0016198, and circ_0005686) interact with miR-877-3p to regulate mitochondrial apoptosis, and the upregulated circRNAs (circ_0002590 and circ_000372) interact with miR-149-5p to inhibit insulin secretion [106,107].

Furthermore, as a class of readily secreted RNA molecules, circular RNAs (circRNAs) have the potential to serve as biomarkers for type 2 diabetes mellitus (T2DM). A study employing RNA sequencing identified 683 differentially expressed circRNAs in blood samples from T2DM patients, with 354 exhibiting upregulation and 329 downregulation. The functional analysis results indicated that these differentially expressed circRNAs may be implicated in apoptotic pathways, suggesting their prospective utility as biomarkers in the near future [108]. Additionally, impaired fasting glucose (IFG) signifies a prediabetic condition; thus, IFG assessment is crucial for alleviating the diabetes burden. Recent evidence indicates that with corresponding area under curves (AUCs) of 0.873 and 0.719 for T2DM and IFG, respectively, a specific set of two circRNAs (hsa_circ_0063425 and hsa_circ_0056891) could effectively differentiate T2DM and IFG patients from healthy controls [109]. Moreover, hsa_circ_0063425 acts as a sponge for miR-19a-3p, while hsa_circ_0056891 serves as a sponge for miR-1-3p; both were positively correlated with AKT and PI3K, respectively [110]. These circRNAs may influence PI3K/AKT signaling pathways to modulate the pathogenesis of T2DM and insulin resistance (Figure 3).

### 4.3. CircRNAs Modulate Insulin Resistance

CircRNA plays a crucial role in the regulation of insulin signaling and lipid metabolism. For instance, circRNA_000203 modulates the expression of the GSK3β gene by targeting miR-26b-5p, thereby influencing the insulin signaling pathway [111]. Furthermore, circRNA_0054633 impacts insulin sensitivity through its interaction with miR-103, which regulates the expression of insulin receptor substrate 1 (IRS1), consequently affecting insulin signal transduction [112].

The functional mechanism of circRNA is primarily contingent upon its cellular localization. Similar to lncRNAs, cytoplasmic circRNAs are widely acknowledged as miRNA decoys. During the process of adipogenesis—encompassing preadipocytes, differentiated preadipocytes, and mature adipocytes—RNA sequencing identified 41 differentially expressed circRNAs, many of which possess binding sites for miRNAs, such as miR-30c, miR-17, and miR-130. This indicates that these circRNAs may play a role in regulating adipogenesis through their potential function as sponge molecules for miRNAs and underscores the critical involvement of circRNAs in adipocyte differentiation and functionality [113]. Notably, the deletion of circH19 was observed to enhance adipogenic differentiation in human adipose-derived stem cells (ADSCs). Mechanistically, the loss of circH19 disrupts its interaction with PTBP1 and accelerates PTBP1’s effect on SREBP1 cleavage and translocation, resulting in an increased expression of genes associated with lipogenesis and lipid accumulation in hADSCs [114]. Furthermore, circSAMD4A facilitates preadipocyte differentiation by sequestering miR-138-5p to elevate EZH2 expression [115]. Adipogenesis is regarded as an intricate process in which multiple regulatory factors and signal pathways are involved. At present, emerging studies demonstrate that circRNAs as regulators of gene expression play crucial roles in adipogenesis and development. Studies suggest that the limitation in adipose tissue plasticity leads to the abnormal functions and metabolism of adipose tissue and drives the progression of lipid-related diseases, like obesity. In this context, circRNAs are emerging as new regulators in adipose tissue plasticity (Figure 3).

## 5. ncRNA-Based Drug Development for T2DM Treatment

The vast majority of the human genome is transcribed into non-coding RNAs (ncRNAs), which do not serve as templates for protein synthesis but play crucial roles in regulating gene expression and influencing disease states [116]. This has positioned ncRNAs as an emerging category of molecular targets in the realm of drug discovery [117]. The unique mechanisms by which ncRNAs exert their regulatory functions present both opportunities and challenges for therapeutic intervention. On one hand, the specificity and versatility of ncRNAs offer a promising avenue for the development of highly targeted treatments [118]. On the other hand, the complexity of their interactions within cellular networks and the lack of well-established methodologies for targeting these molecules introduce significant hurdles [119].

**Figure 4:**
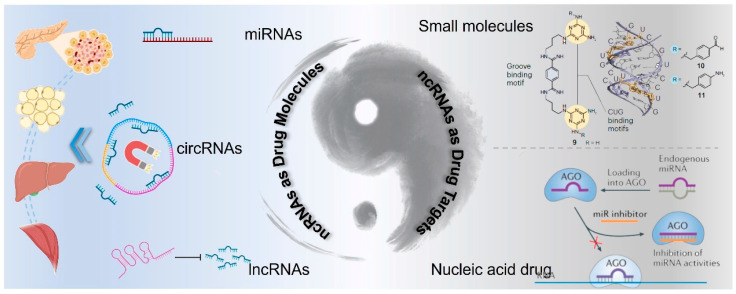
Schematic illustration of ncRNA-based drug discovery: ncRNAs as active drug molecules or drug targets.

Despite these challenges, advances in the technology for targeting messenger RNA (mRNA) provide a foundation upon which strategies for ncRNA-targeted therapies can be built [120]. The expanding body of research on various types of ncRNAs, such as miRNA, circRNAs, and lncRNAs, is elucidating their diverse biological functions and prospects in drug discovery. Here, we discuss the two roles of ncRNAs in drug discovery: as potential active drug molecules or as drug targets, and we further analyze corresponding strategies for ncRNA-based drug development (Figure 4). Moreover, the clinical translation of ncRNA drugs for T2DM treatment is discussed from the perspective of drug delivery.

### 5.1. ncRNAs as Active Drug Molecules

Based on omics analysis techniques, such as transcriptome sequencing, various non-coding RNAs (ncRNAs) with inhibitory activity against type 2 diabetes mellitus (T2DM) have been identified, such as lncRNAs of let7 [121] and miRNAs of miR-802 [38]. These ncRNAs play significant roles in promoting the survival of pancreatic β cells, enhancing insulin sensitivity, and regulating glucose metabolism [122]. Consequently, ncRNAs with therapeutic activity or their mimetics have the potential to be developed as T2DM therapeutic drugs, which can be referred to as ncRNA alternative therapy.

However, at the current stage, the development of ncRNA alternative therapy drugs still faces numerous challenges. Primarily, unlike the siRNA and mRNA drugs currently applied in clinical settings, ncRNAs possess the property of multi-targeting and have relatively poor specificity. Off-target effects can occur when ncRNAs or their mimics interact with unintended targets, leading to unwanted side effects [123]. Careful design of ncRNA sequences and the use of high-throughput screening methods can help identify and minimize off-target effects. Additionally, using specific delivery systems that target particular tissues or cell types can reduce systemic exposure and off-target effects [124]. Developing ncRNA-based therapies requires rigorous preclinical and clinical testing to ensure safety and efficacy. Regulatory approval processes can be lengthy and complex.

Secondly, naked ncRNA molecules are susceptible to degradation by nucleases in the physiological environment, leading to poor stability and bioavailability. Additionally, RNA molecules are negatively charged and large, making it difficult for them to cross cell membranes and enter cells [125]. Chemical modifications can enhance the stability and bioavailability of RNA molecules. Advanced delivery systems, such as lipid-based and polymer-based nanoparticles, can protect RNA molecules and facilitate their cellular uptake [126]. Moreover, the expression and function of ncRNAs can vary among individuals due to genetic and environmental factors, making it challenging to develop universal treatments [127]. Personalized medicine approaches, where treatments are tailored to individual patients based on their ncRNA profiles, can improve therapeutic outcomes. Advances in high-throughput sequencing and computational modeling can facilitate the identification of patient-specific ncRNA targets.

ncRNA mimetic drugs have currently entered the stage of clinical research. RSVI-301, an miRNA alternative therapy developed by RosVivo Therapeutics for insulin resistance, utilizes miRNAs with natural anti-diabetic and anti-obesity properties to act on pancreatic cells, adipocytes, and gastrointestinal cells. By inhibiting the translation of pathogenic genes and restoring the level of miR-10b-5p, it enables the regulation of the miR-10b-5p-mediated KLF11-KIT pathway to reverse the pathology of type 2 diabetes mellitus [128]. According to the research report of RosVivo, RSVI-301 demonstrates superior performance compared to liraglutide/semaglutide, and its side effects are significantly reduced. Additionally, repeated administration of RSVI-301 can reduce insulin resistance to normal levels. Moreover, in terms of patient convenience, the low side effects and long-term efficacy of RSVI-301 are anticipated to exert a major competitive role in the future diabetes market.

### 5.2. ncRNAs as Drug Targets

During the occurrence and development of T2DM, the expression levels of numerous non-coding RNAs (ncRNAs) are pathologically upregulated. Inhibiting the expression and activity of these pathogenic genes holds promise for the treatment of T2DM [129]. Currently, there are two strategies for drug design targeting ncRNAs. (1) Drawing on the experience of developing drugs targeting traditional protein targets, small molecule inhibitors are designed considering the chemical and spatial structural characteristics of ncRNAs [130]. (2) Based on RNA interference technology, single-strand/double-strand oligonucleotides are devised to silence the expression of target ncRNAs [131]. The discovery of these drugs will pioneer new paths for the clinical treatment of T2DM.

#### 5.2.1. Small Molecules Design and Discovery for ncRNA Targeting

To date, nearly all pharmacological agents have been directed towards approximately 700 of the estimated 20,000 disease-associated proteins within the human body [132]. This is primarily attributable to the stable and well-defined conformations typically exhibited by proteins, rendering them suitable candidates for drug targeting. The interaction between a drug and a protein is often analogized to a key fitting into a lock. This precise binding facilitates effective modulation of protein function. In contrast, RNA displays a loose and variable structure characterized by dynamic conformational changes, which significantly hampers the development of therapeutics aimed at RNA targets [133]. Despite these challenges, certain regions within RNA molecules can maintain stable conformations under specific conditions. These structural motifs, including stem loops or conserved secondary structures, show promise as potential therapeutic targets. However, identifying these regions remains complex due to factors such as sequence variability and environmental influences on RNA folding.

In recent years, an expanding body of research has provided compelling evidence that ncRNAs are not merely “junk” but constitute an extensive category of RNA molecules with critical biological functions across various cellular processes. ncRNAs play essential roles in gene regulation, chromatin remodeling, and cellular signaling pathways [134]. For instance, miRNAs can modulate gene expression post-transcriptionally through interactions with target mRNAs while lncRNAs may influence transcriptional activity via interactions with chromatin-modifying complexes [135,136]. Consequently, there has been increasing interest among researchers in developing drugs specifically targeting ncRNAs for therapeutic applications. Compared to the limited number of established protein drug targets, numbering only several hundred, RNA represents an expansive frontier rich with untapped potential for novel therapeutic strategies. As scientists continue to elucidate the diverse roles played by ncRNAs in health and disease contexts, it becomes increasingly evident that they could serve as valuable biomarkers or therapeutic targets [26].

Moreover, advancements in technology have facilitated high-throughput screening methods and computational modeling approaches designed to predict stable RNA structures conducive to drug targeting [137]. Such innovations may pave the way toward overcoming existing barriers associated with therapies aimed at RNA targets [138]. In summary, while significant hurdles remain regarding the identification and validation of stable conformational regions within RNA molecules suitable for pharmacological intervention, ongoing research efforts underscore both their importance in biological systems and their potential utility as innovative drug targets awaiting exploration.

#### 5.2.2. Nucleic Acid Drug Design for ncRNA Targeting

In the current clinical practice, RNA interference (RNAi) technology has been extensively applied in disease treatment strategies targeting mRNA [139]. Globally, 19 small nucleic acid drugs (siRNA, ASO) have been approved for marketing [140]. The therapeutic principle lies in introducing exogenous double-stranded RNA (siRNA) with oligonucleotide fragments (19–23 bp) homologous to the endogenous mRNA coding region into cells, triggering efficient and specific degradation of the corresponding mRNA, thereby resulting in silencing of specific gene expression [141]. The remarkable advantage of RNA interference drugs is that the design and synthesis of complementary oligonucleotides are quite straightforward, especially given that the solid-phase synthesis technology is now mature [142]. In contrast, small molecule drugs targeting specific targets typically require years of lead compound optimization and screening [143]. Another crucial advantage of RNA interference drugs is that they can be utilized to regulate the activity of targets that seem “undruggable” for small molecules, effectively expanding the strategies and ideas for disease treatment [144].

The experience gained from targeting mRNA with oligonucleotides holds significant implications for targeting other RNA species. Currently, the designed and synthesized oligonucleotides have been gradually employed in silencing various types of ncRNAs, offering diverse opportunities and challenges for the discovery of therapeutic drugs for T2DM [145]. Unlike regulating mRNA, ncRNAs can usually regulate multiple downstream target genes. Therefore, blocking a single miRNA may manipulate signaling pathways in multiple dimensions. Additionally, the cellular localization of ncRNAs is more complex than that of mRNA, and it is distributed in both the nucleus and the cytoplasm. Different RNA interference strategies have preferences for the intracellular distribution of target genes. Hence, when choosing RNA interference technologies, the localization of the target nucleic acid should be fully considered. Double-stranded RNA is a reliable approach for targeting RNA in the cytoplasm and inhibiting gene activity. In the nucleus, there are RNAi factors that facilitate the cleavage of the target sequence, indicating that RNAi can also be used to silence ncRNAs distributed within the nucleus. Existing studies suggest that for target genes located in the nucleus, ASO is a more reliable gene silencing agent than double-stranded RNA. Therefore, when the target RNA is located in the nucleus, ASO may be the preferred silencing method; while when the target RNA is located in the cytoplasm, double-stranded RNA is a superior option [146].

In 2021, Thomas Thum demonstrated for the first time in clinical research that the human safety and tolerability of the targeted miRNA drug CDR132L were satisfactory, with no evident toxic responses [147]. Meanwhile, CDR132L significantly reversed the heart failure of patients by inhibiting the function of miR-132 [148]. This study will effectively facilitate the clinical research and development and transformation process of targeted miRNA oligonucleotide drugs. Yu Chen et al. demonstrated that an lncRNA of LINK-A could serve a regulatory function in high-fat diet (HFD)-induced obesity, acting as a pivotal molecular node that modulated the remodeling of the inflammatory microenvironment and energy metabolism within tissue regions [149]. Notably, antisense oligonucleotide (ASO) targeting of LINK-A markedly ameliorated metabolic disorders and obesity symptoms associated with HFD-induced obesity in murine models. The findings suggested that the lncRNA of LINK-A was a promising therapeutic target, offering both theoretical insights and clinical implications for the intervention and management of obesity as well as related metabolic diseases.

### 5.3. Delivery Systems Mobilize Clinical Translation of ncRNA Drugs

Although some non-coding RNA drugs have been approved for disease treatment, since non-coding RNA drugs belong to nucleic acid drugs, like other nucleic acid drugs, the most significant issue for their clinical application is how to deliver non-coding RNA efficiently to the target site [150]. Non-coding RNA drug delivery mainly encounters several problems. Naked single-stranded RNA molecules are readily degraded by nucleases in the physiological environment. RNA molecules possess immunogenicity and can activate the immune system [125]. RNA is a large biomolecule and is negatively charged, making it challenging to cross the cell membrane and enter the cell. RNA molecules have difficulty escaping from endosomes and entering the cytoplasm [151]. Consequently, it is necessary to design appropriate non-coding RNA drug delivery methods or delivery vectors to transport non-coding RNA to the target site so as to fully exploit its tremendous potential for disease treatment. Currently, common non-coding RNA drug delivery systems mainly comprise chemical modification, lipid-based nanocarriers, polymer-based nanocarriers, and some other nucleic acid delivery systems.

#### 5.3.1. Chemical Modifications on RNA Molecules

**Figure 5:**
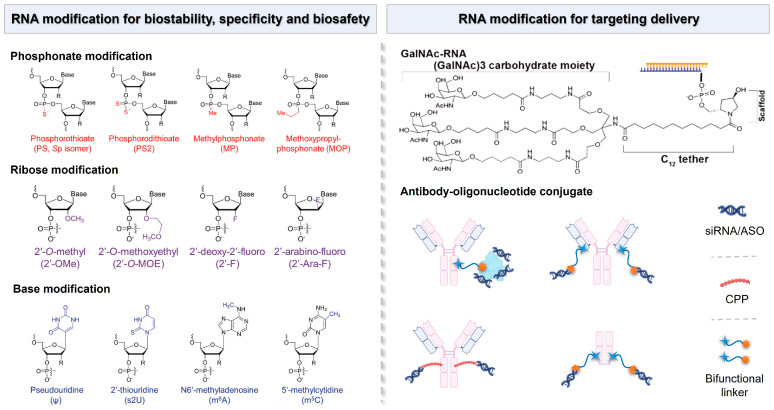
Chemical modifications of RNA molecules for the enhancement of biostability, specificity, and biosafety, as well as the RNA modification strategy for targeting delivery.

Although chemical modification does not directly provide a delivery vector for non-coding RNA drugs, it can significantly enhance the inherent properties of non-coding RNA drugs [152]. Appropriate chemical modifications can improve the physiological stability, immune evasion capacity, and ability to function at the target site of non-coding RNA drugs. For instance, modifications such as 2′-O-methyl, 2′-fluoro, 3′-inverted dT, or polyethylene glycol (PEG) can significantly enhance the serum stability of non-coding RNA and prolong its half-life [153]. Additionally, replacing common nucleic acids with locked nucleic acids (LNAs) where a methylene linkage connects the 2′ and 4′ positions of ribose and substituting the phosphodiester at the 3′ end of the non-coding RNA backbone with thiophosphate can also enhance the drug’s performance. Small molecule 2,4-dinitrophenol (DNP) modification of siRNA not only reduces the degradation of siRNA by nucleases but also facilitates the passage of siRNA through the cell membrane and into the cell (Figure 5) [154].

The nucleic acid-conjugated delivery system involves directly conjugating non-coding RNA to the delivery materials to obtain a well-defined single-component drug delivery system, such as coupling non-coding RNA with polymers, peptides, antibodies, nucleic acid aptamers, or small molecules [155]. This approach enhances the stability and bioavailability of non-coding RNA therapeutics while facilitating targeted delivery to specific tissues or cells [156]. Currently, one of the most promising systems in clinical application is the acetylgalactosamine-siRNA (GalNAc-siRNA)-conjugated delivery system (Figure 5). GalNAc serves as a ligand that specifically binds to hepatic receptors, thereby promoting efficient uptake by liver cells. The trivalent GalNAc-siRNA drugs ALN-TTRsc, ALN-PCS, and ALN-AT3 designed by Alnylam Pharmaceuticals have entered clinical trials and are, respectively, used for treating transthyretin amyloidosis, a condition characterized by misfolded transthyretin protein accumulation and hypercholesterolemia, which involves elevated cholesterol levels, leading to cardiovascular risks and hemophilia, a genetic disorder affecting blood clotting [157].

Inspired by antibody–drug conjugates (ADCs), antibody–siRNA conjugates (ARCs) have emerged as a potential strategy for targeted siRNA drug delivery, and they are capable of overcoming the existing barriers in siRNA delivery (Figure 5) [158]. Currently, researchers have developed methods to couple siRNA with antibodies through non-covalent interactions or covalent linkages with lysine or cysteine residues on the antibody for the treatment of various diseases [159]. Avidity Biosciences, Inc., a pharmaceutical company dedicated to offering the oligonucleotide conjugate platform (AOCs™) for novel RNA therapeutics, announced that the administration of the first patient in the Phase 1/2 clinical study MARINA™ of AOC 1001 has been completed in 2021. The design of AOCs is to integrate the proven monoclonal antibody (mAbs) technology with the precision and potency of oligonucleotide therapeutics for the treatment of previously inaccessible tissue and cell types [160].

These developments highlight not only the versatility of nucleic acid-conjugated systems but also their potential impact on therapeutic strategies for various diseases. Ongoing research aims to optimize these conjugates further through modifications that enhance targeting efficiency and reduce off-target effects. Additionally, understanding the pharmacokinetics and biodistribution profiles of these formulations will be crucial in determining their long-term efficacy and safety in diverse patient populations. As more data from clinical trials become available, they may provide insights into dosage regimens and treatment protocols tailored for individual patients based on specific disease characteristics.

#### 5.3.2. Lipid-Based Nanocarriers

**Figure 6:**
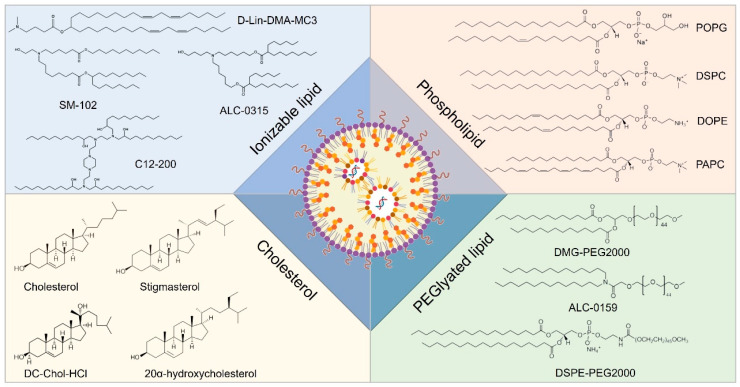
Representative components of LNP formulations including ionizable lipids, sterols, phospholipids, and PEGylated lipids.

Lipid-based nanocarriers have garnered extensive research attention due to their biomimetic architecture and versatile self-assembly characteristics. Based on lipid composition and nanostructure, lipid nanocarriers for RNA delivery can be categorized into lipid nanoparticles, liposomes, lipid nanoemulsions, and solid lipid nanoparticles. Among these, lipid nanoparticles (LNPs) represent the most advanced technology and have been successfully implemented in clinical settings [161]. Three RNA therapeutics utilizing LNPs as carriers have received marketing approval, including Onpattro (patisiran), Comirnaty (BNT162b2), and Spikevax (mRNA-1273). Onpattro (patisiran), an siRNA drug developed by Alnylam Pharmaceuticals, was approved by the Food and Drug Administration (FDA) of the United States in 2018 for the treatment of peripheral neuropathy in adult patients caused by hereditary transthyretin amyloidosis (hATTR). Onpattro is the world’s first siRNA drug to be approved by a regulatory agency. Comirnaty (BNT162b2), an mRNA vaccine jointly developed by Pfizer and BioNTech, is used for the prevention of infection by the novel coronavirus (SARS-CoV-2). At the end of 2020, Comirnaty became one of the first COVID-19 vaccines to obtain emergency use authorization (EUA) worldwide and has subsequently received full approval in multiple countries and regions. Spikevax (mRNA-1273), formerly known as the Moderna COVID-19 Vaccine, is an mRNA vaccine developed by Moderna, also for the prevention of infection by the novel coronavirus. It too received emergency use authorization in several countries in late 2020 and received formal approval subsequently. Typically, LNPs comprise four distinct functional components: ionizable cationic lipids, neutral phospholipids (auxiliary phospholipids), cholesterol, and PEGylated lipids [162]. The advancement of ionizable cationic lipids is pivotal for the clinical translation of LNPs. Conventional quaternary ammonium structural cationic lipids, such as DOTAP, maintain a consistent positive charge; while they efficiently load negatively charged nucleic acid molecules like RNA through electrostatic interactions, this often results in high positive charges [163]. Consequently, the resultant nanoparticles exhibit a pronounced positive zeta potential within the bloodstream, leading to significant nonspecific adsorption of plasma proteins and immune system activation that facilitates their clearance from circulation. Ionizable cationic lipids predominantly feature tertiary amine structures that are electrically neutral under physiological pH conditions, thereby exhibiting low cytotoxicity; however, they become positively charged in the acidic milieu of endocytic vesicles at lower pH levels. This property aids in promoting the escape of drug-encapsulated LNPs from endosomal/lysosomal compartments [164]. Auxiliary phospholipids play a crucial role in enhancing the assembly of LNP nanostructures while ensuring structural stability. Cholesterol contributes rigidity to the molecular structure by not only augmenting LNP stability but also improving fusion capabilities with target cell membranes. PEGylated lipids facilitate the formation of a stable hydration layer on LNP surfaces, which mitigates serum protein adsorption as well as non-specific cellular uptake and immune clearance, thus extending systemic circulation time for LNPs (Figure 6) [165].

**Figure 7:**
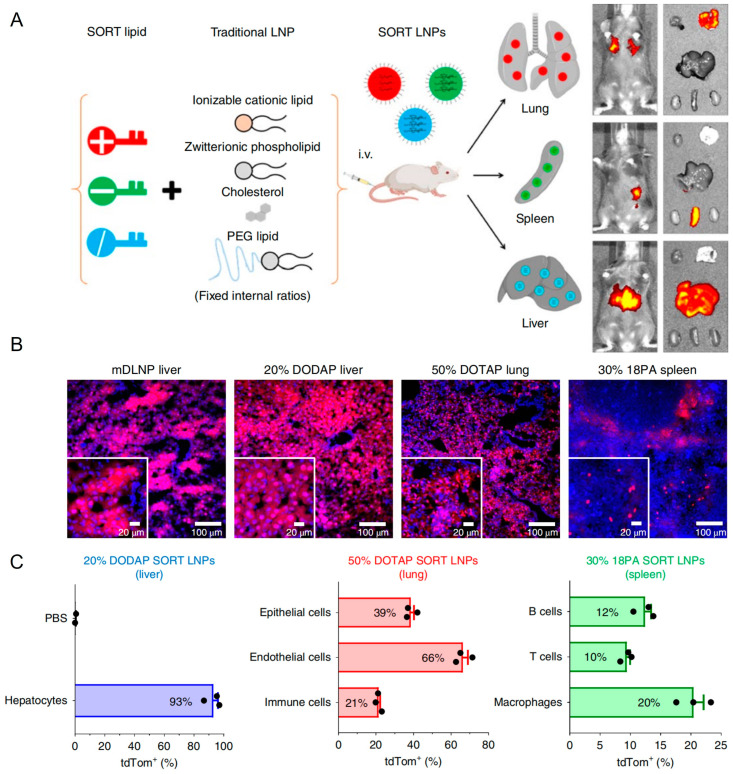
(**A**) The addition of a supplemental component (termed a SORT molecule) to traditional LNPs systematically alters the in vivo delivery profile and mediates tissue-specific delivery as a function of the percentage and biophysical property of the SORT molecule. This methodology successfully redirected multiple classes of nanoparticles. (**B**) Delivery of Cre mRNA activates tdTom expression in tdTom transgenic mice via Cre-mediated genetic deletion of the stop cassette. Confocal microscopy was employed to further verify effective tissue editing (n = 3 biologically independent animals). (**C**) FACS was used to quantify the percentage of tdTom+ cells within defined cell type populations of the liver, lung, and spleen (0.3 mg kg^−1^, day 2). Data are presented as mean ± s.e.m. (n = 3 biologically independent animals).

Research efforts aimed at optimizing LNP functionality primarily focus on achieving targeted delivery beyond hepatic tissues. Currently, available clinically developed LNP formulations inherently exhibit spontaneous targeting towards liver tissues via apolipoprotein-mediated adsorption within the plasma, which is a limitation that significantly constrains their therapeutic application across various diseases. Qiang Cheng et al. introduced selective organ targeting (SORT) technology by incorporating charge-regulating lipid components into LNP formulations, enabling efficient lung- and spleen-targeted delivery outcomes (Figure 7A) [166]. As illustrated in Figure 7B,C, SORT LNPs contributed to high transfection in targeting organs, which suggested extensive prospects in clinical application and gene drug development. In treating T2DM, ncRNA drug targets predominantly include islet β cells or adipose tissue. Therefore, exploring targeted delivery technologies for pancreatic and adipose tissue-directed administration using LNPs could substantially enhance therapeutic efficacy. Jilian R. Melamed described an LNP formulation that potently and selectively delivered mRNA to the pancreas. The researchers showed that intraperitoneal delivery of mRNA-LNPs induces robust protein expression in the pancreas for structurally distinct ionizable lipids. This strategy induced protein expression primarily in β cells, which were insulin-producing cells located in pancreatic islets. Furthermore, they showed that mRNA delivery was facilitated by peritoneal macrophage extracellular vesicle (EV) transfer and that efficacy was not linked to systemic toxicity. Together, these data suggested that mRNA-LNPs were a viable, non-viral means of inducing protein expression in difficult-to-transfect pancreatic cells [167]. Similar delivery strategies could be further designed for ncRNA delivery to islet β cells.

Adipose-targeted delivery strategies can be developed based on the unique physiological architecture of adipose tissue, including its blood vessels, extracellular matrix, and cell-surface-specific receptors. Kang Chen et al. modified an adipose tissue-homing peptide (CKGGRAKDC) on the surface of lipid nanoparticles to facilitate efficient delivery of thyroid hormone by specifically recognizing statins that are highly expressed in adipose tissue vasculature, thereby effectively enhancing adipose metabolism, reversing obesity, and preventing the onset of type 2 diabetes mellitus (T2DM) [168]. During obesity progression, the expansion of adipose tissue is accompanied by an increase in the extracellular matrix (ECM), which contains glycosaminoglycans that are known as some of the most negatively charged biological macromolecules, thereby enabling the effective trapping of positively charged nanoparticles within adipose tissue [169]. Yunxiao Zhang et al. employed cationic nanoparticles for the targeted delivery of rosiglitazone (RSG) to adipose tissues in conjunction with photothermal therapy to significantly promote browning processes in white adipose tissue and markedly ameliorate diet-induced obesity [170]. Furthermore, scavenger receptors are abundantly expressed on the surface cells within adipose tissues; thus, lipoprotein-like structures can be utilized for high-efficiency targeting. Junhua Han et al. utilized recombinant high-density lipoprotein to deliver resveratrol, a known activator of fat browning, to enhance metabolic states in obese mice while achieving a remarkable reduction of 68.2% in inguinal white adipose mass [171].

#### 5.3.3. Polymer-Based Nanocarriers

Polymeric carriers represent the most extensively researched vehicles for nucleic acid drug delivery in preclinical settings, offering advantages such as excellent biocompatibility, biodegradability, safety, non-toxicity, and potential for surface modification [172]. Cationic polymers are predominantly utilized as polymeric carriers for nucleic acid delivery. Polyethyleneimine (PEI) serves as a quintessential example of cationic polymers and is capable of forming linear or branched molecular structures with varying molecular weights. The 25 kDa branched PEI is widely regarded as the “gold standard” for gene transfection due to its favorable properties. Rich in primary and secondary amines, PEI readily undergoes protonation in aqueous solutions and possesses a high density of positive charges that facilitate efficient compression of nucleic acid drugs into nanoparticles [173]. Upon cellular uptake of PEI nucleic acid (NA) nanoparticles, the acidic lysosomal environment triggers the “proton sponge” effect inherent to PEI, enabling rapid lysosomal escape and resulting in enhanced transfection efficiency. The stability of high molecular weight PEI poses challenges regarding degradation and metabolism in vivo, potentially leading to accumulation toxicity [174,175,176], while small molecule variants exhibit insufficient gene compression capacity. To address this limitation, researchers have conjugated small molecules with cleavable chemical structures, such as disulfide bonds and borate esters, to create degradable high molecular weight PEIs. This innovative approach combines the effective nucleic acid compression capabilities characteristic of large molecular weight PEIs, with rapid degradation facilitated by intracellular microenvironments, accelerating nucleic acid drug release while generating small peptide fragments that promote metabolic clearance and enhance carrier safety. Furthermore, excess positive charge associated with conventional cationic carriers often results in cytotoxicity [177]. Therefore, modifications targeting N-containing groups within PEI have been explored to neutralize its positive charge under specific microenvironmental triggers. For instance, phenylborate derivatives designed by our group can effectively convert cationic polymers into neutral or negatively charged forms upon reactive oxygen species (ROS) stimulation, thereby facilitating the burst release of nucleic acids. The strategy could improve both transfection efficiency and overall safety profiles associated with cationically modified polymer carriers.

To further augment the safety profile of cationically charged polymers, extensive research has focused on natural cationic materials alongside synthetic analogs mimicking natural compounds. Chitosan (CS), a natural amino polysaccharide characterized by low cost, biodegradability, good biocompatibility, and minimal immunogenicity, stands out among these candidates. The CS polymer exhibits positive charge under acidic conditions, allowing efficient electrostatic interactions necessary for compressing nucleotides into nanostructures. Nevertheless, the transfection efficacy of CS remains constrained, primarily due to limited charge density solubility issues [178]. Consequently, modifications, like methylating amino groups, could improve solubility and charge densities. For instance, trimethylchitosan demonstrates significantly enhanced performance compared with traditional CS polymers. Additionally, natural basic amino acids, including lysine, arginine, and histidine, possess intrinsic positive electricity, rendering them ideal substrates for constructing safe biodegradable polycation systems. Polylysine (PLL), the most prevalent form of polyamino acid, not only possesses superior compatibility but also exhibits a remarkable ability to mediate successful transfection of various types of genetic drugs (siRNA plasmid DNA) both in vitro and in vivo. Thus, polyamino acids, like PLL polymers, share promising clinical translation prospects in gene drug development. At the current stage, the polymer-based nanocarriers have been advanced to clinical research [179]. Notably, StemiRNA Therapeutics has developed a lipopolyplex (LPP) system for RNA drug delivery. In the LPP structure, negatively charged RNA is compressed by positively charged polypeptides to form a nanocomplex, followed by the encapsulation of lipid bilayers yielding core–shell architectures [180]. Recently, an LPP-based RNA drug has been approved for further clinical trials.

#### 5.3.4. Other Delivery System for ncRNA Drugs

Extracellular vesicles (EVs) serve as crucial pathways for intercellular communication, transporting a diverse array of biomolecules, including non-coding RNAs (ncRNAs), to target organs [181]. They play a significant role in the onset and progression of diabetes and its complications by modulating insulin sensitivity and β cell functionality. Emerging evidence suggests that extracellular vesicle-derived ncRNAs are pivotal regulators in the development and advancement of diabetes [182]. Iacopo Gesmundo et al. discovered that EVs enriched with miR-155-5p derived from obese human adipose tissue resulted in β cell death and dysfunction [183]. Bin Qian et al. reported that M1 macrophages and islet-resident macrophages of obese mice secreted exosomes enriched with miR-212-5p, leading to impaired β cell insulin secretion [184]. Ting Liu et al. evidenced that obese adipose tissue macrophage-derived exosomes (ATM-Exos) encapsulating miR-29a could be transferred into myocytes, causing insulin resistance [185]. Consequently, leveraging endogenous ncRNA trafficking pathways presents a potentially effective strategy for achieving efficient delivery of therapeutic active ncRNAs. Compared to synthetic drug carriers, extracellular vesicles offer multiple advantages as natural carriers; their endogenous origin and membrane proteins confer an extended half-life in vivo. Furthermore, they demonstrate superior delivery efficiency with reduced cytotoxicity compared to conventional transfection methods.

Inorganic nanoparticles have gained prominence in nucleic acid delivery and imaging research due to their highly controllable structures, uniform dispersion, and diminutive particle sizes. Gold nanoparticles, silica nanoparticles, and iron oxide nanoparticles are commonly used in gene drug delivery, among which gold nanoparticles and iron oxide variants have been utilized clinically and are generally regarded as non-toxic nanomaterials [186]. Gold nanoparticles exhibit unique optical properties alongside ease of synthesis and surface functionalization capabilities that allow selective co-modification with nucleic acids through covalent or non-covalent conjugation techniques. Nucleic acid chains can be covalently linked to the core of gold nanoparticles (typically 13–15 nm) via thiol groups. The strategy is applicable for both DNA and siRNA and can be directly attached either to bare gold cores or polymer-modified counterparts. Rafael A. Vega et al. conjugated oligonucleotides on gold nanoparticles, and the oligonucleotide-modified AuNP could mediate efficient transfection for islets. The transfected islets produced a 100% diabetes cure rate after transplantation [187]. Chengming Jia et al. used gold nanoparticles to deliver miR155 antagonists for the treatment of diabetic cardiomyopathy. After tail vein injection, antago-miR15-conjugated gold nanoparticles could preferentially transport the nucleic acids into the macrophages via phagocytosis. Together with the increased M2 ratio and reduced inflammation, in vivo delivery of antago-miR155 reduced cell apoptosis and restored the cardiac functions of diabetic mice [188]. The surfaces of these gold nanoparticles may also be encapsulated by alternating layers of anionic nucleic acids with polycations, such as PEI or polyamidoamine (PAMAM), along with targeting ligands, facilitating specific interactions with cell surface receptors [189]. Iron oxide nanoparticles composed primarily of Fe_3_O_4_ or Fe_2_O_3_ possess superparamagnetic properties at nanoscale dimensions; they have demonstrated efficacy as delivery vehicles as well as agents for heat-based therapies. Most existing delivery systems utilize surface-engineered cationic iron oxide nanoparticles that engage electrostatically with anionic nucleic acid therapeutics. For instance, lipid-coated iron oxide nanoparticles ranging from 50 to 100 nm could exhibit optimal activity for siRNA delivery and transfection [190].

## 6. Conclusions

In conclusion, the emerging roles of non-coding RNAs (ncRNAs) in the pathogenesis and therapeutic strategies of type 2 diabetes mellitus (T2DM) underscore their significant potential as biomarkers and therapeutic targets. As a chronic and severe non-communicable disease, T2DM imposes a considerable burden on global health systems, characterized by elevated blood glucose levels and intricate metabolic abnormalities. The complex interplay among various types of ncRNAs, including miRNAs, lncRNAs, and circRNAs, along with their regulatory mechanisms in cellular processes provides novel insights into the molecular foundations of T2DM. Preclinical studies have demonstrated the promising efficacy of innovative delivery systems such as oligonucleotide nanoparticles (ONPs), RNAi microsponges, and exosome-based platforms in enhancing stability, bioavailability, and targeted delivery of ncRNA therapeutics. These delivery systems not only improve pharmacokinetic properties but also mitigate off-target effects, thereby enhancing therapeutic precision. For instance, miRNA mimics and antagomirs have shown promise in modulating key pathways involved in insulin resistance and β cell dysfunction—suggesting their utility in restoring metabolic homeostasis. Moreover, the therapeutic potential of ncRNAs extends beyond direct modulation of gene expression; circRNAs and lncRNAs can function as “sponges” to sequester miRNAs—thereby indirectly regulating multiple target genes’ expression. This multifaceted regulatory network offers a robust framework for developing combination therapies that address T2DM’s heterogeneous nature. Additionally, advancements in ncRNA-based diagnostics, such as liquid biopsies, could facilitate early detection alongside personalized treatment strategies—further improving patient outcomes. Despite these advancements, several challenges persist. The complexity inherent to ncRNA interactions within cellular networks necessitates more precise and efficient delivery methods—a critical area for future research endeavors. Addressing these challenges will require interdisciplinary collaboration that integrates expertise from molecular biology to nanotechnology through clinical medicine. Furthermore, large-scale clinical trials are essential for validating both safety and efficacy regarding ncRNA-based therapies across diverse patient populations. In summary, exploring ncRNAs within T2DM not only broadens our understanding but also paves the way for discovering new diagnostic tools alongside therapeutic interventions. By leveraging unique properties inherent to ncRNAs, we can develop more targeted treatments—ultimately contributing to improved patient outcomes while enhancing quality of life. Future research should continue focusing on unraveling intricate mechanisms underlying ncRNA dysregulation while translating findings into clinically viable strategies.
